# Association of Allergic Conditions with Adolescent Sleep Duration: A National Survey

**DOI:** 10.3390/children12101356

**Published:** 2025-10-09

**Authors:** Hyeseon Choi, Eunju Seo, Jinju Woo

**Affiliations:** 1 College of Nursing, Woosuk University, Wanju 55338, Republic of Korea; hschoi@woosuk.ac.kr; 2 Department of Nursing, Paichai University, Daejeon 35345, Republic of Korea; 3 Department of Nursing, Kyungnam University, Changwon 51767, Republic of Korea

**Keywords:** allergic rhinitis, asthma, eczema, sleep duration, adolescent, complex sampling design

## Abstract

**Highlights:**

**What are the main findings?**

- Allergic rhinitis was significantly associated with shorter weekday sleep duration among Korean adolescents.
- Asthma and eczema were not significantly associated with sleep duration.

**What is the implication of the main finding?**

- Allergic rhinitis management may be essential in improving sleep health among adolescents.
- Future studies should examine objective physiological measures of sleep to complement self-reported data.

**Abstract:**

Background: Allergic diseases, such as allergic rhinitis, eczema, and asthma, are prevalent among adolescents and are associated with various health concerns, including poor sleep quality and mental health problems. Although previous research has investigated the general association between allergic conditions and sleep disturbances, few studies have examined how allergic diseases relate to sleep duration. Methods: We performed secondary analysis of the data obtained from the 19th Korea Youth Risk Behavior Survey (2023), which included 52,880 middle and high school students. Data was analyzed using complex sample design techniques, descriptive statistics, t-tests, and analyses of variance and covariance conducted to explore associations between allergic diseases and sleep duration on weekdays. Covariates included sex, school type, academic performance, socioeconomic status, and residential type. Results: The average weekday sleep duration among adolescents was 6.2 h, which was significantly shorter than that recommended by the U.S. Centers of Disease Control and Prevention (8–10 h). Among allergic conditions, allergic rhinitis was significantly associated with reduced sleep duration (*p* = 0.001), unlike asthma (*p* = 0.119) and eczema (*p* = 0.586). Additional differences in sleep duration were observed by sex, academic performance, socioeconomic status, and living arrangements. Conclusions: Managing allergic rhinitis may be crucial to promoting adequate sleep during adolescence. Furthermore, future research should incorporate physiological indicators to assess sleep quality, as self-reported measures may not capture sleep disturbances such as night-time awakenings. These findings can inform the development of integrated health strategies to enhance physical and psychological well-being of adolescents.

## 1. Introduction

Allergic diseases are one of the most common conditions that make daily living uncomfortable and can range from mild symptoms of nasal congestion, sneezing, and a runny nose to severe cases of anaphylaxis [1]. According to the World Health Organization, the prevalence of allergic diseases is increasing worldwide, and in Korea, the prevalence of allergic rhinitis and atopic dermatitis is particularly high in metropolitan areas [2,3].

Allergic diseases are caused by a combination of factors, which can be both genetic and environmental [4,5,6,7]. These allergic conditions include allergic rhinitis, atopic dermatitis, and asthma, which are associated with mental health problems in adolescence, including psychological anxiety, depression, suicidal ideation and attempts [8], and high rates of physical inactivity and school absenteeism [9], which can affect learning. Allergic diseases are associated with various factors, which can be both genetic and environmental [4,5,6,7]. These allergic conditions include allergic rhinitis, atopic dermatitis, and asthma, which are also associated with mental health problems in adolescence, including psychological anxiety, depression, suicidal ideation and attempts [8], and high rates of physical inactivity and school absenteeism [9]. All of these can impact learning.

Sleep is one of the most important factors in maintaining daily functioning, and its quality and quantity are important. The quality and quantity of sleep can vary depending on the individual; however, on average, the appropriate amount of sleep during adolescence is between 8 and 10 h [10]. Korean adolescents sleep very little, with 6 h of sleep for middle school students and 6 h for high school students [11]. Sleep deprivation in adolescents has been linked to various mental health problems, such as depression, anxiety, and cognitive decline [12]; thus, managing sleep during adolescence is considered essential.

In existing research on the effect of allergic conditions, allergic conditions in adolescents are associated with poor sleep quality and reduced sleep satisfaction [13]. However, these studies have included allergic rhinitis in the definition of rhinitis, and no studies have distinguished the relationship between sleep duration and different types of allergic diseases, such as allergic rhinitis, atopic dermatitis, and asthma. In allergic rhinitis, as well as atopic dermatitis and asthma, understanding how symptoms such as itching and breathlessness affect sleep is crucial. Early assessment and intervention for these sleep problems can help prevent and manage further physical and mental health problems. Previous studies have shown that allergic conditions, allergic conditions in adolescents are associated with poor sleep quality and reduced sleep satisfaction [13]. However, these studies have categorized allergic rhinitis under the broader definition of rhinitis, Furthermore, no reports have examined the relationship between sleep duration and different types of allergic diseases, such as allergic rhinitis, atopic dermatitis, and asthma. Previous studies have reported that allergic conditions are associated with poor sleep quality and decreased sleep satisfaction in adolescents [14,15]. However, no studies have separately analyzed the relationship between sleep duration and individual allergic diseases such as allergic rhinitis, atopic dermatitis, and asthma [14,16]. Understanding the association between each disease on respiratory function during sleep is essential for prompt assessment and intervention [14,15] and ultimately the prevention of future physical and psychological health problems [17]. Accordingly, this study aimed to determine the degree of allergic disease morbidity and sleep duration in adolescents, extent of sleep duration according to the general characteristics of adolescents, and the relationship between sleep duration and the presence and type of allergic disease. The findings will provide a basis for proper sleep management and allergic disease management in adolescents. We hope that this study can be used as a basis for proper sleep management for adolescents in the future.

## 2. Methods

### 2.1. Study Design

This study is a secondary analysis of the big national data from the 19th Korea Youth Risk Behavior Survey in 2023, aimed at investigating the relationship between allergic diseases and sleep duration among Korean adolescents [18].

### 2.2. Sample and Participants

The target study population consisted of middle and high school students enrolled in South Korea as of April 2023. The 19th Korea Youth Risk Behavior Survey (2023) was conducted across a total of 799 schools (399 middle schools and 400 high schools), including 52,880 students (28,401 middle school students and 24,479 high school students). Students who had been absent for extended periods, those unable to participate independently due to severe disabilities, and students with reading difficulties were excluded [18].

### 2.3. Measurements

#### 2.3.1. Weekday Sleep Duration

Weekday sleep duration was measured based on responses to the question, “What time do you usually go to bed and wakeup during the past 7 days?” Participants provided their usual bedtime (hour and minute) and wakeup time (hour and minute) for weekdays (Monday to Friday) through an online self-report survey [18].

#### 2.3.2. Subjective Sleep Satisfaction Rate

Subjective sleep satisfaction was evaluated based on the response to the question, “Over the past 7 days, do you feel that the amount of sleep you had was sufficient for recovering from fatigue?” Responses were categorized as “very adequate,” “adequate,” “not enough,” “so-so,” and “not getting enough at all” [18].

#### 2.3.3. Asthma Diagnosis by a Physician

Asthma diagnosis was determined based on responses to the question, “In the past 12 months, have you been diagnosed with asthma by a physician?” Responses were categorized as “no” or “yes” [18].

#### 2.3.4. Allergic Rhinitis Diagnosis by a Physician

In this study, allergic rhinitis diagnosis was determined based on responses to the question, “In the past 12 months, have you been diagnosed with ‘allergic rhinitis’ by a physician?” Responses were categorized as “no” or “yes” [18].

#### 2.3.5. Eczema Diagnosis by a Physician

Eczema diagnosis was determined based on responses to the question, “In the past 12 months, have you been diagnosed with ‘eczema’ by a physician?” Responses were categorized as “no” or “yes” [18].

### 2.4. Data Collection and Ethics

This study analyzed secondary data from the 19th Korea Youth Risk Behavior Survey (2023), which was conducted by the Korea Disease Control and Prevention Agency (KDCA) after receiving national approval (No. 117058). The original survey was approved by the KDCA Institutional Review Board, (IRB), and data collection was carried out performed following strict ethical procedures, including anonymous, voluntary participation via mobile devices. In this study, we used only de-identified, publicly available secondary data. Therefore, our data use was exempted from further ethical review by the Kyungnam University IRB (IRB No. 1040460-E-2024-001), in accordance with national guidelines on secondary data analysis.

Sampling was conducted using a stratified cluster sampling method by KDCA. The primary and secondary sampling units were schools and classes, respectively. For the primary sampling, schools were selected using a stratified random sampling technique. For the secondary sampling, one class per grade was randomly chosen from the selected schools. Starting in 2023, the 19th Korea Youth Risk Behavior Survey was conducted using mobile devices (e.g., tablets and smartphones), that is, the survey support teachers distribute one student information sheet per student, explaining the need for and method of participation—either through a video presentation or PowerPoint instructions. In this study, in classrooms with Internet access, the survey supports teachers provided each student with a mobile device for anonymous participation. Students accessed the survey system using a participation number printed on their information sheet, voluntarily agreed to participate, and completed the survey anonymously [18].

### 2.5. Data Analysis

This study utilized data from the Korea Youth Risk Behavior Survey after data cleaning (including logical error correction and outlier treatment), weight generation, and stratification integration. All analyses were conducted using IBM SPSS Statistics version 27 (IBM Corp., Armonk, NY, USA), with significance set at α = 0.05 for the two-tailed test.

The analysis considered the complex sampling design by incorporating strata, weights, cluster variables, and finite population correction. Descriptive statistics, including frequencies, percentages, means, and standard deviations, were used to summarize the general characteristics and variables related to sleep and allergic conditions. Differences in sleep duration by participant characteristics were evaluated using complex sample t-tests or analysis of variance. The relationship between sleep duration and allergic conditions was analyzed using analysis of covariance within a generalized linear model to account for the complex sampling design.

## 3. Results

### 3.1. Participant Characteristics

The general characteristics of the study participants are presented in Table 1.

### 3.2. Adolescents’ Sleep and Allergy Disease Characteristics

The average weekday sleep duration among Korean adolescents was 6.2 h. The most common self-reported sleep satisfaction level was “not enough” (33.6%), whereas the least common was “very adequate” (7.8%). Over the past 12 months, the highest proportion of adolescents diagnosed with allergic diseases was for allergic rhinitis (68.9%), followed by eczema (49.7%) and asthma (35.3%) (Table 2).

### 3.3. Differences in Weekday Sleep Duration Among Adolescents

Significant difference was noted in sleep duration per week among the study participants based on sex (t = 15.92, *p* < 0.001), school type (t = 52.46, *p* < 0.001), academic grades (F = 63.74, *p* < 0.001), economic status (F = 63.19, *p* < 0.001), living arrangements (F = 17.67, *p* < 0.001), and allergic rhinitis (t = 2.08, *p* = 0.001).

Female students had significantly less sleep during the weekdays than male students. High school students also slept less during the weekdays than middle school students. Students with lower academic grades slept less during the weekdays. Conversely, adolescents with better economic status and those living with their parents slept more during the weekdays. However, adolescents diagnosed with allergic rhinitis slept less (Table 3).

### 3.4. Relationship Between Allergic Disease and Weekday Sleep Duration

To examine the relationship between the diagnosis of allergic diseases (asthma, allergic rhinitis, and eczema) and weekday sleep duration among Korean adolescents, sex, school type (middle school and high school), academic grades, economic status, and living arrangements were adjusted as covariates, and the results were analyzed, as shown in Table 4.

In this study, adolescents diagnosed with allergic rhinitis (*p* = 0.001) had significantly shorter sleep during the week than those without this condition. However, adolescents diagnosed with asthma (*p* = 0.119) and eczema (*p* = 0.586) did not show a significant difference in weekday sleep duration compared with their nonallergic peers (Table 4).

## 4. Discussion

In this study, the average weekday sleep duration among adolescents was 6.2 h, which fell significantly short of the 8–10 h recommended by the U.S. Centers for Disease Control and Prevention (CDC) [19]. In addition, the proportion of adolescents who subjectively reported their sleep as “not enough” was the highest at 33.6%. Insufficient sleep among adolescents may lead to various adverse outcomes, including increased anxiety, depression, and decreased academic performance [12]. Insufficient sleep duration can exacerbate adolescents’ depression, anxiety, and reduced academic performance through increased daytime napping and impaired concentration [20]. In fact, the number of medical consultations for depression and anxiety disorders among children and adolescents in South Korea in 2022 increased by 113.9% and 136.6%, respectively, compared with 2018 data. Moreover, the adolescent suicide rate was 2.13 per 100,000 population as of 2019, which is higher than the Organization for Economic Co-operation and Development (OECD) average of 1.70 and ranks within the top 10 among OECD countries [21]. Allergic rhinitis showed the highest 12-month prevalence among physician-diagnosed allergic diseases, followed by eczema and asthma. The global prevalence of allergic diseases has been increasing; this trend is often attributed to urbanization and environmental pollution [22]. Furthermore, these conditions have been associated with anxiety disorders and depression among adolescents. Given the strong associations with sleep deprivation, obesity, smoking, and psychological stress, allergic conditions may need continuous and systematic management [21,23]. In the present study, allergic conditions were assessed using self-reported items that evaluated physician diagnosis and reference periods (ever and/or during the past 12 months). The survey also allowed respondents to report multiple allergic conditions. Furthermore, because the KYRBS is school based, the timing of data collection (academic term and season) may differ from those of other international surveys. Accordingly, direct cross-survey comparisons should be interpreted cautiously.

Regarding sex differences in sleep duration, female students reported shorter weekday sleep than their male counterparts. This outcome may be attributed to earlier wakeup times, higher levels of academic stress and responsibility, and greater sensitivity to social expectations and psychological demands commonly observed among female adolescents [24]. These findings are consistent with the results of previous studies, thereby supporting the present results [25,26].

In examining differences in sleep duration by school grade, middle school students were found to have longer sleep than high school students. This may be attributed to the increased academic burden, heightened stress associated with university entrance preparation, and greater participation in private tutoring among high school students, which often results in extended study hours late into the night and consequently shorter sleep [27,28]. This interpretation is supported by a previous study indicating that participation in private tutoring increases significantly after the third year of middle school [28]. These findings align with the results of a previous study suggesting that sleep duration progressively declines as students reach higher-grade levels [29].

Furthermore, students with higher academic achievement tended to have longer sleep than those with lower academic performance. This may be attributed to their greater self-regulation abilities, which enable them to maintain consistent sleep patterns. These findings are consistent with the results of previous studies suggesting that longer sleep is associated with better academic outcomes [27,30]. Conversely, students with lower academic performance may sleep less because of high stress levels or maladaptive sleep habits such as going to bed late [31].

No significant association was found between parents’ educational level and adolescents’ sleep duration. This finding implies that factors such as parenting style and household rules may serve as more influential mediating variables than parental education [32]. However, some studies have reported contrasting findings; for instance, adolescents with more educated parents had shorter sleep durations than those whose parents had not completed high school [33]. Considering that the study referenced included a racially diverse sample, it differs from the present study, which concentrated on students in South Korea, thereby pointing to the need for further research in culturally specific contexts.

Consistent with these findings, previous studies have indicated that adolescents with lower socioeconomic status tend to experience shorter sleep durations and report more subjective sleep problems [34]. Furthermore, children living in neighborhoods characterized by lower socioeconomic status have also been found to sleep less [35], thereby reinforcing the results of the present study. Differences in sleep duration according to socioeconomic status were also noted. Adolescents from lower socioeconomic backgrounds demonstrated shorter sleep durations, whereas those from higher socioeconomic backgrounds reported longer sleep durations. This finding may be attributed to the fact that economically disadvantaged households often encounter poor living conditions, emotional instability, and inadequate sleep environments, which may negatively affect sleep. Conversely, adolescents from wealthier families may benefit from more stable and comfortable conditions conducive to adequate sleep [34].

Adolescents residing in protective facilities such as orphanages, social welfare centers, and group homes are less likely to participate in supplementary learning activities, such as private tutoring or after-school academies, compared with their peers living at home. Along with this characteristic, the relatively structured and restricted living environment of protective facilities may help maintain more stable sleep patterns. Furthermore, higher residential satisfaction has been associated with longer sleep duration [36,37]. Taken together, these findings indicate that adolescents living in protective facilities may more consistently achieve a certain level of sleep duration. However, this interpretation remains tentative, and further empirical research is needed to confirm it.

These findings indicate that adolescents’ sleep duration is associated not only by individual factors but also by a complex interplay of social and environmental determinants. Therefore, ensuring healthy sleep patterns among adolescents requires not only adjustments in academic schedules at the school level but also concerted efforts to improve sleep-related environments within families and local communities.

An analysis of the relationship between allergic diseases and sleep duration revealed that adolescents diagnosed with allergic rhinitis had shorter weekday sleep durations than those without the condition. This finding proposes that the symptoms of allergic rhinitis may be negatively associated with both the quality and quantity of sleep. Specifically, allergic rhinitis is characterized by nasal congestion, sneezing, and rhinorrhea, which often worsen at night and consequently reduce sleep duration [38]. Although the difference (0.1–0.2 h; 6–12 min) appears modest, the weekly cumulative lose (30–60 min across school nights) may meaningfully shift the proportion of adolescents below recommended sleep thresholds (i.e., <8 h). Thus, even small individual effects can be relevant from a public health perspective.

During adolescence, getting enough sleep is essential for academic success, emotional stability, and physical development. Therefore, sleep deprivation associated with allergic rhinitis may negatively associated with the physical and psychological well-being of adolescents. Moreover, inadequate sleep may worsen symptoms of allergic rhinitis [13], highlighting the bidirectional nature of this relationship. Therefore, managing allergic rhinitis effectively in adolescents is not only crucial for improving sleep quality but may also contribute to reducing the severity of allergic rhinitis.

Sleep duration among adolescents with asthma or eczema did not significantly differ from that of their peers without these conditions. Asthma is characterized by nocturnal exacerbations and dyspnea [39]. In line with the findings of the present study, although the sleep onset and wakeup times of adolescents diagnosed with asthma were like those without asthma, they may experience sleep fragmentation or reduced sleep depth because of asthma symptoms during the night. Indeed, a previous study also showed that adolescents with asthma do not necessarily exhibit shorter sleep durations than those without asthma [40]. However, several studies have reported that asthma may impair sleep quality rather than sleep duration [41].

In addition, when asthma symptoms are mild or well-controlled through medication, nocturnal asthma attacks may not occur, which could explain the lack of significant difference in sleep duration [42]. Similarly, in the case of atopic dermatitis, a previous study found that adolescents diagnosed with this condition do not have significantly shorter sleep durations than those without it [43]. However, as with asthma, eczema also negatively affects sleep quality [43]. Furthermore, when asthma symptoms are mild or well-controlled through medication, nocturnal asthma attacks are less likely to occur, which could explain the lack of significant difference in sleep duration [42]. Similarly, in the case of atopic dermatitis, a previous study found that adolescents diagnosed with this condition do not have significantly shorter sleep durations than those without it [43]. However, as with asthma, eczema is also negatively associated with sleep quality [43].

Furthermore, sleep duration was measured using a self-report method based on questions about sleep onset and wakeup times. As a result, sleep interruptions during the night were not captured. Therefore, to more accurately evaluate sleep in adolescents with asthma or atopic dermatitis, future studies are recommended to include both physiological indicators and subjective assessments of sleep quality. This would enable a better understanding of how these conditions may influence aspects of sleep other than total sleep duration.

### Limitation

Weekday sleep duration and allergic conditions were self-reported, rendering the measures susceptible to recall bias and misclassification. Such errors, particularly for mild or past diagnoses, may be non-differential, likely biasing estimates toward a null result. Nevertheless, the KYRBS’s standardized protocols and the large, nationally representative sample may help mitigate such random errors.

In addition to measurement error, the incomplete adjustment for key determinants warrants consideration. The analysis did not systematically adjust for key potential confounders, including mental health (e.g., depressive symptoms, perceived stress), screen time, and lifestyle factors (caffeine use, physical activity, smoking/alcohol. This oversight may have resulted in residual confounding, with the possibility of under- or overestimating the true associations. Future research should employ more precise measurements in analyzing these variables, which may help mitigate residual confounding.

Because the national dataset did not include items that assessed sleep quality or disease control levels (e.g., severity, symptom frequency, medication use), additional analyses in these respects were not possible. This limitation restricts the interpretation of our findings and highlights the need for future studies incorporating more detailed clinical indicators.

Finally, our findings are based on a nationally representative sample of Korean adolescents and may not be generalized to adolescents in other countries who may experience different social, cultural, and academic pressures. Contextual features such as school schedules, private tutoring, and examination intensity, may influence sleep behaviors in population-specific ways. Accordingly, the estimates should be interpreted cautiously as context-dependent associations rather than universally applicable effects. Furthermore, future studies examining more diverse settings are needed to establish comparability and generalizability.

## 5. Conclusions

In this study, the association between allergic diseases and sleep duration among adolescents was investigated by analyzing the relationship between the two variables. The findings revealed that adolescents’ average weekday sleep duration was significantly lower than the recommended levels suggested by the CDC.

Notably, adolescents diagnosed with allergic rhinitis reported shorter sleep durations on weekdays, suggesting a potential negative association between allergic rhinitis and sleep in this population. Conversely, no significant differences in sleep duration were found among adolescents diagnosed with asthma or eczema, indicating that these conditions may not directly influence total sleep time.

To promote healthy sleep among adolescents, a systematic approach to the management of allergic diseases is necessary. Given the strong association between allergic rhinitis and reduced sleep duration, active medical intervention, increased social awareness, and supportive management within educational settings are recommended.

Additionally, sleep quality must also be addressed. As this study relied on a self-reported questionnaire based on sleep onset and wakeup times, it had limited ability to capture night-time awakenings or comprehensively evaluate sleep quality. Future research should incorporate physiological measures to assess sleep more accurately and develop effective strategies for improving sleep quality among adolescents with allergic conditions.

The findings of this study may contribute to the development of strategies aimed at enhancing the physical and psychological well-being of adolescents.

## Figures and Tables

**Table 1:** Participants’ general characteristics.

Characteristics	Categories	n ^1^	% ^2^
Sex	Male	26,769	51.5
	Female	26,111	48.5
School type	Middle school	28,401	50.9
	High school	24,479	49.1
Academic grades *	High	20,041	38.1
	Medium	15,540	29.4
	Low	17,294	32.5
Father’s educational level *	Less than or equal to middle school graduate	489	1.2
	High school graduate	8433	23.1
	College graduate or higher	26,039	75.7
Mother’s educational level *	Less than or equal to middle school graduate	397	1.0
	High school graduate	9339	25.2
	College graduate or higher	26,203	73.9
Economic status *	High	22,410	43.5
	Medium	23,981	44.8
	Low	6484	11.7
Living arrangements *	Living with the family	50,362	95.9
	Living with relatives	293	0.6
	Boarding, living independently (including living with friends)	358	0.6
	Dormitory	1643	2.5
	Care facilities (orphanage, social welfare facility, and group home)	217	0.4

(N = 52,880); ^1^ Unweighted number. ^2^ Weighted percentage. * Exclude missing values.

**Table 2:** Sleep and allergy disease characteristics.

Characteristics	Categories	n ^1^	% ^2^
Sleep *			
Sleep time (h)	Weekday sleep duration ^3^	6.24 ± 0.01	
Subjective sleep	Very adequate	4209	7.8
Satisfaction rate	Adequate	9802	18.3
	Not enough	17,793	33.6
	So-so	14,696	28.0
	Not getting enough at all	6380	12.4
Allergic disease *			
Asthma	No	1813	64.7
	Yes	1002	35.3
Allergic rhinitis	No	6076	31.1
	Yes	13,298	68.9
Eczema	No	5726	50.3
	Yes	5701	49.7

^1^ Unweighted number. ^2^ Weighted percentage. ^3^ Weighted mean ± standard error. * Exclude missing values.

**Table 3:** Differences in weekday sleep duration by participant characteristics.

Characteristics	Categories	Sleep Duration (h)		t or F	*p*
		M ^1^	SE ^2^		
Sex	Male	6.48	0.02	15.92	<0.001
	Female	5.99	0.02		
School type	Middle school	6.77	0.01	52.46	<0.001
	High school	5.69	0.01		
Academic grades *	High	6.33	0.02	63.74	<0.001
	Medium	6.22	0.02		
	Low	6.15	0.02		
Father’s educational level *	Less than or equal to middle school graduate	6.14	0.09	1.040	0.354
	High school graduate	6.16	0.04		
	College graduate or higher	6.13	0.04		
Mother’s educational level *	Less than or equal to middle school graduate	6.11	0.10	1.15	0.318
	High school graduate	6.14	0.03		
	College graduate or higher	6.18	0.03		
Economic status *	High	6.32	0.02	63.19	<0.001
	Medium	6.21	0.01		
	Low	6.07	0.02		
Living arrangements *	Living with the family	6.25	0.01	17.67	<0.001
	Living with relatives	6.03	0.09		
	Boarding, living independently (including living with friends)	5.93	0.10		
	Dormitory	5.85	0.06		
	Care facilities (orphanage, social welfare facility, group home)	6.59	0.14		
Allergic disease *					
Asthma	No	5.92	0.07	−0.08	0.934
	Yes	5.93	0.09		
Allergic rhinitis	No	6.05	0.10	2.08	0.038
	Yes	5.81	0.06		
Eczema	No	5.91	0.07	−0.41	0.685
	Yes	5.95	0.09		

^1^ Unweighted number. ^2^ Weighted percentage. * Exclude missing values.

**Table 4:** Relationship between allergic disease and weekday sleep duration.

Variables	Categories	Adjusted Mean	95% CI		Difference (Yes—No)	95% CI	B	SE	t	*p*
			Lower	Upper						
Asthma	No	6.09	6.02	6.15	−0.09	−0.02–0.20	0.09	0.06	1.56	0.119
	Yes	6.00	5.91	6.09						
Allergic rhinitis	No	6.18	6.15	6.22	−0.07	0.03–0.11	0.07	0.02	3.30	0.001
	Yes	6.11	6.09	6.14						
Eczema	No	6.11	6.07	6.14	0.01	−0.07–0.04	−0.01	0.03	−0.54	0.586
	Yes	6.12	6.08	6.16						

## Data Availability

The data that support the conclusions of this article can be found in the Korea Youth Risk Behavior Web-based Survey repository at https://www.kdca.go.kr/yhs/home.jsp (accessed on 30 October 2024).

## Abbreviations

The following abbreviations are used in this manuscript:

CDC	U.S. Centers for Disease Control and Prevention
IRB	institutional review board
OECD	Organization for Economic Co-operation and Development
